# *Lentinula Edodes Mycelia* extract regulates the function of antigen-presenting cells to activate immune cells and prevent tumor-induced deterioration of immune function

**DOI:** 10.1186/s12906-023-04106-5

**Published:** 2023-08-08

**Authors:** Shota Kajiyama, Takahiro Nagatake, Satoru Ishikawa, Koji Hosomi, Yuki Shimada, Yasunori Matsui, Jun Kunisawa

**Affiliations:** 1 Laboratory of Vaccine Materials and Laboratory of Gut Environmental System, Microbial Research Center for Health and Medicine, National Institutes of Biomedical Innovation, Health and Nutrition (NIBIOHN), 7-6-8 Asagi Saito, Ibaraki-city, Osaka 567-0085 Japan; 2 Graduate School of Pharmaceutical Sciences, Osaka University, Osaka, Japan; 3 Central R & D Laboratory, Kobayashi Pharmaceutical Co., Ltd, Ibaragi, Osaka Japan; 4 Laboratory of Functional Anatomy, Department of Life Sciences, School of Agriculture, Meiji University, Kanagawa, Japan; 5 Department of Microbiology and Immunology, Kobe University Graduate School of Medicine, Kobe, Japan; 6 International Vaccine Design Center, The Institute of Medical Science, The University of Tokyo, Tokyo, Japan; 7 Graduate School of Medicine, Graduate School of Dentistry, Graduate School of Science, Osaka University, Suita, Japan

**Keywords:** Dendritic cells, Macrophages, Antigen-presenting cells, Immunotherapy, *Lentinula Edodes*

## Abstract

Immune cell activation is essential for cancer rejection; however, the tumor microenvironment leads to deterioration of immune function, which enables cancer cells to survive and proliferate. We previously reported that oral ingestion of *Lentinula Edodes Mycelia* (L.E.M.) extract enhances the tumor antigen-specific T-cell response and exerts an antitumor effect in a tumor-bearing mouse model. In this study, we focused on antigen-presenting cells (APCs) located upstream of the immune system, induced a T-cell response, then examined the impact of L.E.M. extract on the APCs. L.E.M. extract enhanced the expression of MHC-I, MHC-II, CD86, CD80, and CD40 in bone marrow-derived dendritic cells (DCs) and strongly induced the production of IL-12. L.E.M.-stimulated DCs enhanced IFN-γ production from CD8^+^ T cells and induced their differentiation into effector cells. Furthermore, L.E.M. extract promoted IL-12 production and suppressed the production of IL-10 and TGF-β by transforming bone marrow-derived macrophages into M1-like macrophages. Furthermore, in a B16F10 melanoma inoculation model, DCs in the spleen were decreased and their activation was suppressed by the presence of cancer; however, ingestion of L.E.M. extract prevented this functional deterioration of DCs. In the spleen of cancer-bearing mice, the number of CD11b^−^ F4/80^+^ macrophages in a hypoactivated state was also increased, whereas L.E.M. extract suppressed the increase of such macrophages. These findings suggest that L.E.M. extract may exhibit an antitumor immune response by regulating the function of APCs to induce cytotoxic T lymphocytes, as well as by suppressing the decline in antigen-presenting cell activity caused by the presence of cancer.

**Supplementary Information:**

The online version contains supplementary material available at 10.1186/s12906-023-04106-5.

## Introduction

The immune system is the most important mechanism preventing the invasion of foreign enemies and maintaining homeostasis in the human body; it also functions as a biological defense mechanism against infectious diseases such as viruses and bacteria [1]. Furthermore, the immune system plays an important role in eliminating cancer by identifying cancer cells generated in the body as abnormal [2]. Thus, it is important for each step of the cancer immunity cycle to function in order to eliminate cancer cells efficiently [3]. When antigen-presenting cells (APCs) capture cancer antigens, they increase the expression of major histocompatibility complex (MHC) and auxiliary signaling molecules such as CD80, CD86, and CD40 [3, 4], and migrate to the T-cell region of the regional lymph nodes by passing through lymph vessels in a CCR7-dependent manner [5]. In lymph nodes, APCs present antigens to naïve CD4^+^ T cells via MHC-II, then release various cytokines such as IL-12, IL-10, and IL-4 to promote their differentiation into effector T cells such as Th1 cells and Th2 cells [3]. Furthermore, APCs induce cytotoxic T lymphocyte (CTL) responses by presenting the antigen to naïve CD8^+^ T cells via MHC-I [6]. Activated CTLs infiltrate and eliminate the cancer cell, then release more cancer antigens, thereby repeating the cycle [3]. Thus, to eliminate cancer, APCs must first capture and activate cancer antigens. Specifically, dendritic cells (DCs) and macrophages present antigens to T cells and induce potent T-cell activation, making these APCs crucial for cancer elimination [6–8].

The immune surveillance system, which includes efficient activation of immune cells and local infiltration of CTLs into tumors, is a major mechanism in cancer elimination [2]. However, it is also important to understand the cancer immune escape system, which generates an environment where cancer cells escape immune surveillance, thereby favoring the growth of cancer cells [7]. Cancer cells eliminate cancer antigens and escape recognition from immune cells through the expression of immune checkpoint molecules such as PD-L1, and produce various immunosuppressive factors such as TGF-β, IL-6, and VEGF to suppress the activation of immune cells such as DCs, macrophages, and T cells [9, 10]. Furthermore, an environment that helps cancer cells escape immunity is constructed through the mobilization of immunosuppressive cells such as myeloid-derived suppressor cells, tumor-associated macrophages, and regulatory T cells in the cancer microenvironment [11].

Against this background, research is being conducted on substances that can activate immune cells and disrupt the immunosuppressive condition by cancer-bearing hosts [3, 9–11]. For example, *Lentinula edodes mycelia* (L.E.M.) extract, which is made by culturing shiitake mushrooms in a medium consisting of bagasse and rice bran, extracting them with boiling water before germination, and pulverizing them, is a dry powder containing various components such as organic acids (e.g. syringic acid and vanillic acid) and polysaccharides (e.g. α-glucan and arabinoxylan-like polysaccharides) [12, 13]. In our previous study, we used cancer-bearing mouse models, including a B16F10 melanoma inoculation model and CT-26 colorectal cancer inoculation model, to show that L.E.M. extract intake induces tumor antigen-specific CTLs and decreases the proportion of regulatory T cells [14, 15]. Furthermore, in a peptide vaccine administration model using the B16 antigen, the combination of L.E.M. extract intake and vaccine administration strongly enhanced the B16 antigen-specific T-cell response and suppressed tumor growth compared with administration of the vaccine alone [16]. The ingestion of L.E.M. extract also suppresses the production of TGF-β derived from T cells and IL-6 in the blood [14–17]. In addition, a study using standardized L.E.M. (a mixture of polysaccharides, amino acids, lipids, and minerals) produced by culturing in liquid medium reported prolonged tumor development time, increased number of NK cells, and increased IFN-γ-producing tumor antigen-specific CTLs in the B16F0 melanoma inoculated mouse model [18]. Furthermore, a study using a mouse model inoculated with MC38 colon cancer cells reported reduced tumor growth and increased expression of granzyme B and Ki-67 by tumor-infiltrating CTLs in the combination of standardized L.E.M. group compared to the group in which PD-1 and CTLA-4 were inhibited simultaneously [19]. Thus, L.E.M. extract intake is useful for enhancing antitumor immunity by inducing an antitumor response with CTL generation. Nevertheless, it remains unclear how L.E.M. extract induces these CTLs. Therefore, in this study, we investigate the effects of L.E.M. extract on the APCs involved in T-cell response.

## Materials and methods

### Experimental mice

C57BL/6 N female mice (4–7 weeks old) were purchased from Japan SLC inc. (Shizuoka, Japan). These mices were reared by housing in a SPF-controlled room at 22 ℃. The animals were acclimatized to laboratory conditions for seven days prior to the start of the experiments. The animals were euthanized by cervical dislocation under isoflurane anesthesia (FUJIFILM Wako Pure Chemical, Osaka, Japan).

### Preparation of L.E.M. reagents

As previously discussed, L.E.M. dry powder was obtained by culturing shiitake fungus in a medium consisting of bagasse and rice bran, then extracting with boiling water and pulverizing it immediately prior to germination when the hyphae spread in the medium [10]. L.E.M. extract was used in an appropriate concentration for each experiment by adjusting it with phosphate buffered saline (PBS).

### Chemical profile of L.E.M. extract

High performance liquid chromatography (HPLC) was performed using a HPLC prominence (Shimazu, Kyoto, Japan). The conditions for HPLC analysis are as follows: columns; Atlantis T3 column (3 μm, 150 mm × 3 mm) (Waters, Milford, MA, USA), column temperature; 40 °C, flow rate; 0.5 mL/min, injection volume; 1.0 µL, wavelength; 190–800 nm (photo diode array); mobile phase; a mixture of water (A) and acetonitrile (B). The eluent gradients were set as A-B (vol/vol) from 90:10 to 5:95.

### Generation and culture of bone marrow-derived DCs or macrophages

Bone marrow-derived DCs (BMDCs) were prepared from bone marrow (BM) of C57BL/6 N mice as described previously [20]. Briefly, BM cells were treated with 1X RBC Lysis Buffer (Thermo Fisher Scientific, Inc., Waltham, MA, USA) for 1 min then washed with PBS. The cells were then cultured in an RPMI medium containing 20 ng/mL GM-CSF (PeproTech, Cranbury, NJ, USA) and RPMI 1640 (Sigma-Aldrich, St. Louis, MO, USA), to which 10% FBS (Thermo Fisher Scientific, Inc.) and 1% penicillin-streptomycin mixed solution (FUJIFILM Wako Pure Chemical), 1 mM sodium pyruvate solution (Nacalai Tesque, Inc., Kyoto, Japan), and 55 nmol/L mercaptoethanol (FUJIFILM Wako Pure Chemical) were added; half of the medium was replaced with fresh medium every two days. On Day 6, BMDCs were purified according to the manufacturer’s protocol using a CD11c microbeads (Miltenyi Biotech, Bergisch Gladbach, Germany) magnetic separation system (MACS; Miltenyi Biotech). BMDCs (1.25 × 10^4^ cell/well) were seeded to a 96-well plate. We then added various concentrations of L.E.M. extract or lipopolysaccharide (LPS) (Sigma-Aldrich) for positive control before culturing in the RPMI-1640 medium for 12 h. After culture, the supernatant and cells were recovered. The effect of endotoxin contamination of L.E.M. extract was evaluated by adding 5 µg/mL of polymyxin B sulfate (FUJIFILM Wako Pure Chemical) to BMDCs.

BM-derived macrophages (BMDMs) were obtained as previously described [21, 22]. Simply, the BM cells were cultured in Repcell (CellSeed Inc., Tokyo, Japan) using DMEM culture medium (10% FBS, 1% penicillin-streptomycin mixed solution, 55 µmol/L mercaptoethanol) containing 50 ng/mL of M-CSF (PeproTech); half of the medium was replaced with fresh medium every two days. On Day 6, the floating cells were washed and removed, and the cells adhered to the bottom of the plate were recovered as BMDMs. BMDMs (2.0 × 10^5^ cell/well) were seeded to a 24-well plate and cultured for three days in DMEM medium, to which 20 ng/mL of IFN-γ (PeproTech) was added to induce M1-like macrophages. Similarly, BMDMs were seeded with 20 ng/mL of IL-4 (PeproTech) to induce M2-like macrophages. When inducing M1-like macrophages or M2-like macrophages, we added L.E.M. extract or LPS for positive control to them. After culture, the cells were recovered. Similarly, BMDMs were seeded to a 24-well plate, L.E.M. extract was added, and the mixture was cultured for three days in DMEM medium, to which IFN-γ or IL-4was added. After culture, the cells were stimulated for 6 h with LPS (10 ng/mL) and the supernatant was recovered. Furthermore, M1-like macrophages or M2-like macrophages were induced from BMDMs. Next, we added either IL-4 plus L.E.M. extract to the M1-like macrophages or IFN-γ plus L.E.M. extract to the M2-like macrophages, then cultured the mixture for three days in DMEM medium. Each set of macrophages was stimulated for 6 h with LPS after culture, and the supernatant was recovered.

### Co-culture of BMDCs with T cells

We collected the spleens of seven-week-old C57BL/6 N female mice, and separated naïve CD4^+^ T cells and CD8^+^ T cells in the spleen using the CD4^+^CD62L^+^ T-Cell isolation kit II (Miltenyi Biotec) and CD8α (Ly-2) MicroBeads, mouse (Miltenyi Biotec) according to the manufacturer’s protocol for the magnetic separation system. The separated naïve CD4^+^ T cells (2 × 10^5^ cells/well) and CD8^+^ T cells (2 × 10^5^ cells/well) were seeded to a 96-well plate containing 100 µL of 3 µg/mL anti-mouse CD3ε antibody (BioLegend, San Diego, CA, USA) that had been allowed to solidify overnight, along with BMDCs (1 × 10^5^ cells/well) that were either unstimulated or stimulated with L.E.M. extract (10 µg/mL) or LPS (10 ng/mL, as a positive control) for 12 h, then cultured for five days in RPMI-1640 medium (10% FBS, 1 mM sodium pyruvate solution, 1% penicillin-streptomycin mixed solution, 55 nmol/L mercaptoethanol). After culture, the supernatant and cells were recovered.

### Flow cytometry analysis

Flow cytometry analysis was conducted as previously described [15]. First, cells were stained with anti-CD16/32 monoclonal antibody (Thermo Fisher Scientific) to prevent non-specific staining. Next, the dead cells were stained with 7-AAD (BioLegend) or Zombie Green Fixable Viability Kit (Biolegend), as well as with the monoclonal antibodies (mAbs) described below, then analyzed using MACSQuant (Miltenyi Biotec) or BD Accuri C6 (BD Biosciences, San Jose, CA, USA). The mAbs used for staining were shown in Supplementary Table 1. The data were analyzed using FlowJo LLC Software 10.2 (BD Biosciences).

### Measurement of cytokines

IL-12, IL-10, TGF-β, and IFN-γ were quantified using an IL-12 p70 Mouse Uncoated ELISA Kit (Thermo Fisher Scientific), IL-10 Mouse Uncoated ELISA Kit (Thermo Fisher Scientific), TGF beta-1 Human/Mouse Uncoated ELISA Kit (Thermo Fisher Scientific), and IFN gamma Mouse Uncoated ELISA Kit (Thermo Fisher Scientific), respectively, according to the manual of each kit.

### Tumor cell lines and in vivo antitumor assay

The B16F10 mouse melanoma cell line derived from C57BL/6J mice was purchased from ATCC (ATCC number: CRL-6475). A total of 7.5 × 10^5^ B16F10 cells were injected subcutaneously into the left sole of the mice. On day 7 after administering the tumor cells, the mice were orally administered 200 µL of L.E.M. extract daily at a dose of 1 g /kg or 2 g/kg. To evaluate the degree of tumor regression, 120 mg/kg gemcitabine (GEM) (Tokyo Chemical Industry Co. Ltd, Tokyo, Japan) was administered intraperitoneally twice on days 10 and 17 as a positive control. On day 28, the mice were euthanized, the tumor weight was measured, and the spleen was recovered. The tumor size was measured twice weekly, and calculated as 0.5 × length × width^2^ (mm^3^). The recovered spleen was then washed by treating the cell suspension with a 1X RBC Lysis Buffer for 1 min after passing it through a 100-µm cell strainer. Finally, the cell subset in the spleen was analyzed by flow cytometry.

### Statistical analysis

Data were evaluated using the unpaired two-sided Student’s t-test, the two-sided Welch’s t-test or one-way ANOVA and Tukey-Kramers post-hoc tests. A result of *p* < 0.05 was regarded as significant.

## Results

### Chemical profile of L.E.M. extract

A chemical profile of L.E.M. extract was shown in Supplementary Fig. 1. L.E.M. extract has been reported to contain diverse components, especially syringic acid and vanillic acid, which are among the richest in mushrooms bed extracts [12, 13]. The L.E.M. extract used in this study also contains 650 µg/g of syringic acid and 1000 µg/g of vanillic acid, which might be responsible for biological activities of L.E.M. extract [23, 24].

### L.E.M. extract activates BMDCs

**Fig. 1:**
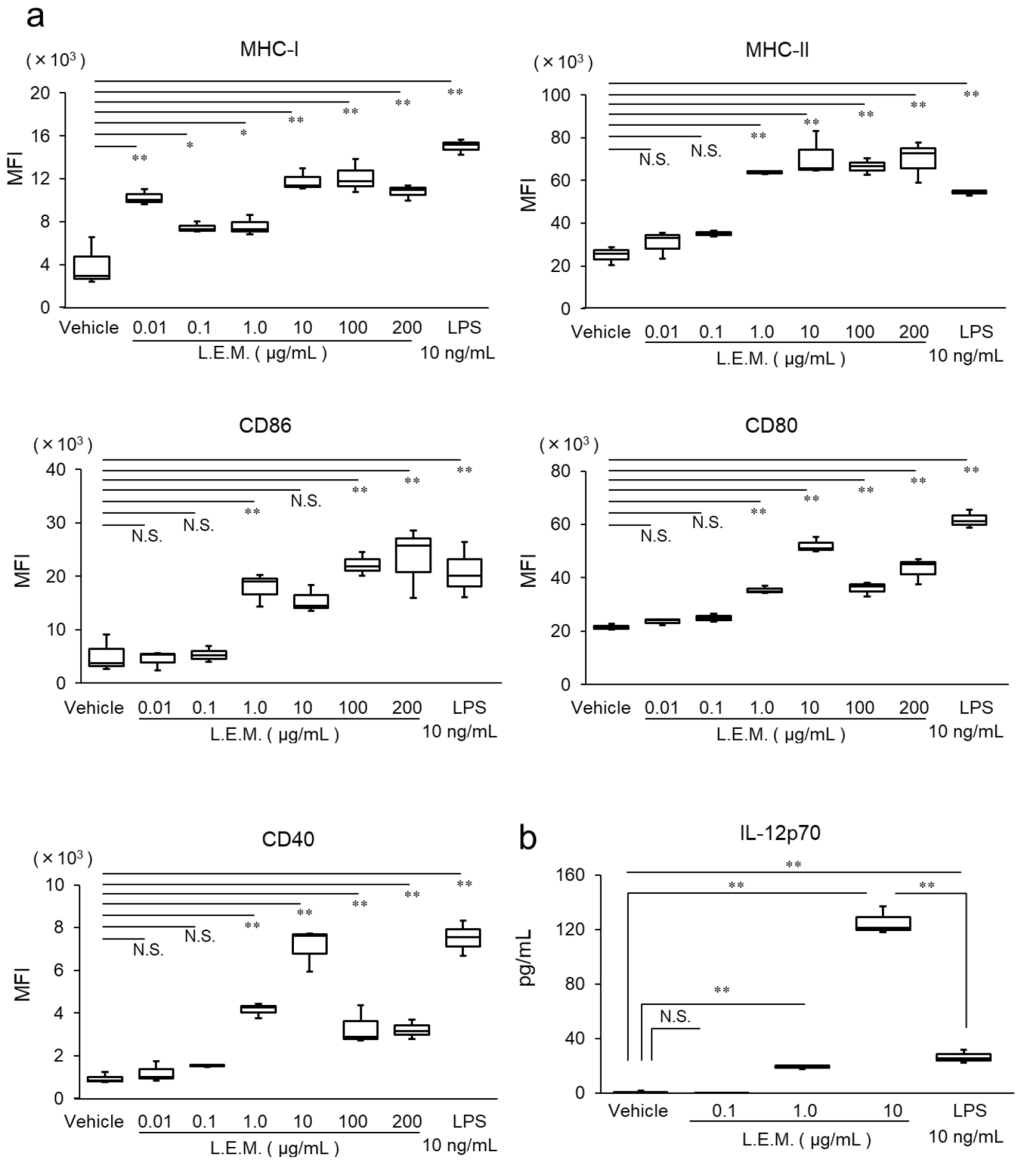
L.E.M. extract directly activates BMDCs. BMDCs were stimulated by either solvent, 0.01, 0.1, 1, 10, 100, or 200 µg/mL L.E.M. extract, or 10 ng/mL LPS (a positive control). After 12 h of incubation, the expression levels of (**a**) MHC-I, MHC-II, CD80, CD86 and CD40 were analyzed by flow cytometry (N = 3 per group). The median value is shown in the figure. The data are representative of two independent experiments and were statistically analyzed by one-way ANOVA (*, p < 0.05, compared to vehicle; **, p < 0.01, compared to vehicle; N.S., not significant, compared to vehicle). (**b**) ELISA measurement of IL-12p70 production in the culture supernatant (N = 3 per group). The median value is shown in the figure. The data are representative of two independent experiments and were statistically analyzed by one-way ANOVA (*, p < 0.05; **, p < 0.01; N.S., not significant)

To examine the effects of L.E.M. extract on the activation of DCs, we first induced BMDCs from mouse BM and performed flow cytometry to measure the expression levels of MHC-I and MHC class II, and co-stimulators CD80, CD86, and CD40 when cells were stimulated by L.E.M. extract and LPS. We observed that L.E.M.-stimulated BDMCs causing a dose-dependent and significant increase in the expression levels of MHC-I, MHC-II, CD80, CD86, and CD40, and that LPS-stimulated BMDCs also increased their expression levels (Fig. 1a). Furthermore, L.E.M.-stimulated BMDCs dose-dependently enhanced the production of IL-12p70, which is the Th1 cell-inducing cytokine [25]. LPS-stimulated BMDCs also produced IL-12p70; however, at doses where MHC-II, CD80, CD86, and CD40 expression levels were comparable to those when stimulated with L.E.M. extract, IL-12p70 production was lower than that when stimulated with L.E.M. extract (Fig. 1b). When polymyxin B (5 µg/mL) was added to BMDCs to investigate possible endotoxin contamination of L.E.M. extracts, the addition of polymyxin B significantly decreased the expression levels of MHC-I and CD86 in the presence of LPS (10 ng/mL), whereas L.E.M. extracts (10 µg/mL) addition, no change was observed (Supplementary Fig. 3). These data suggest that L.E.M. extract may directly activate BMDCs by a different mechanism to LPS and contribute to the activation of T cells such as Th1 cells and CTLs.

### BMDCs activated by L.E.M. extract promote T-cell activation

**Fig. 2:**
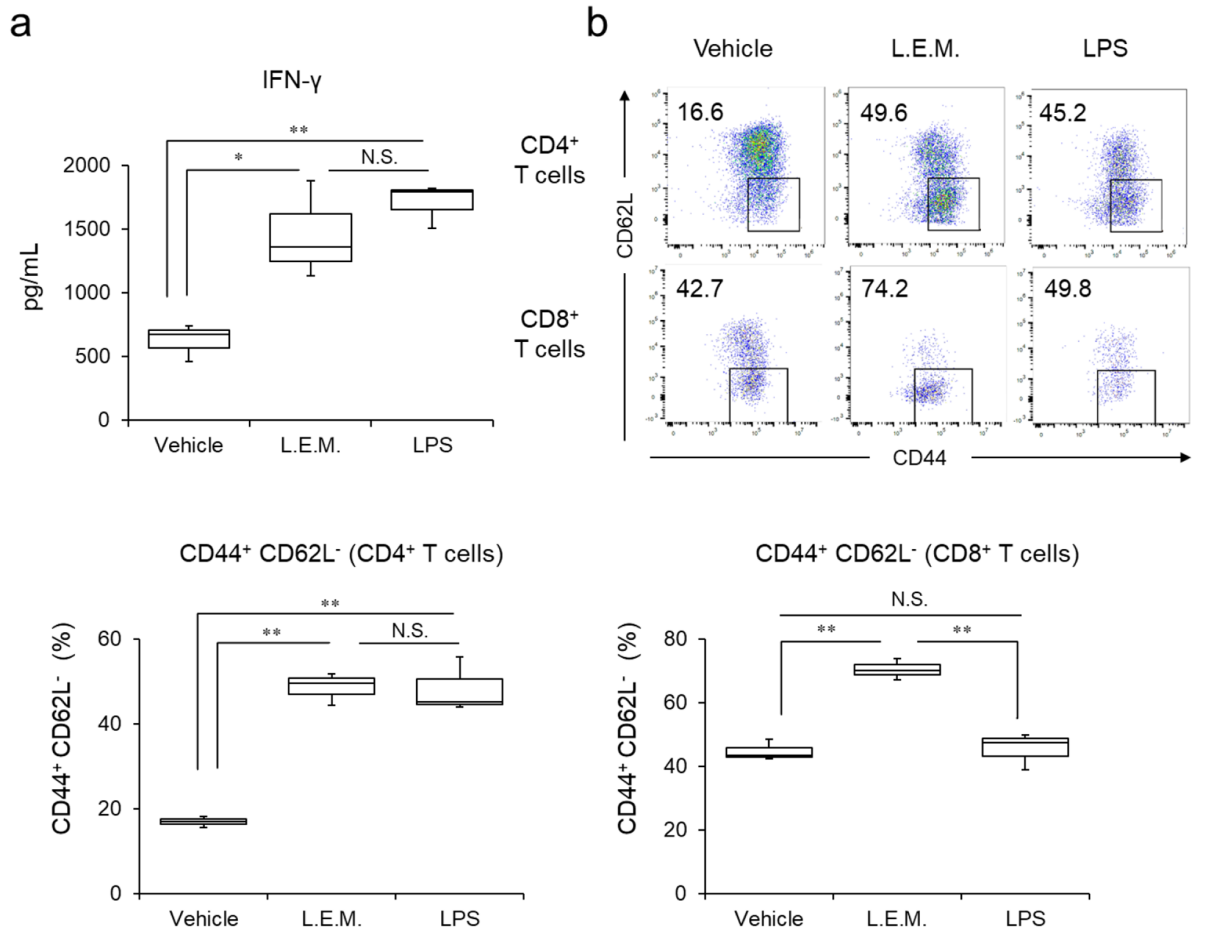
L.E.M. extract-stimulated BMDCs promote T-cell activation. CD4^+^ T cells and CD8^+^ T cells were isolated from spleens using the MACS system and co-cultured for five days with BMDCs stimulated by 10 µg/mL L.E.M. extract or 10 ng/mL LPS (a positive control) for 12 h. (**a**) IFN-γ in CD8^+^ T-cell culture supernatant measured by ELISA assay (N = 3 per group). (**b**) Proportion of cells expressing CD44 and CD62L analyzed by flow cytometry (N = 3 per group). The numbers show the percentage of CD62L^−^CD44^+^ cells and typical flow cytometry results. The median value is shown in this figure. The data are representative of two independent experiments and were statistically analyzed by one-way ANOVA (*, p < 0.05; **, p < 0.01; N.S., not significant)

First, CD8^+^ T cells were isolated from the spleen using MACS beads, and BMDCs stimulated with L.E.M. extract or LPS were co-cultured for five days under stimulation with anti-CD3ε antibody. Second, IFN-γ in the supernatant was measured by ELISA assay. The results showed that L.E.M.- and LPS-stimulated BMDCs significantly increased IFN-γ production compared to the unstimulated BMDCs (Fig. 2a). Furthermore, flow cytometry was used to analyze the functional changes of T cells when naïve CD4^+^ T cells and CD8^+^ T cells were isolated and co-cultured with L.E.M.- or LPS-stimulated BMDCs. The proportion of CD44^+^CD62L^−^ effector memory T cells increased in CD4^+^ T cells and CD8^+^ T cells when co-cultured with L.E.M.-stimulated BMDCs was higher than that when co-cultured with unstimulated BMDCs (Fig. 2b). Conversely, LPS-stimulated BMDCs increased CD44^+^CD62L^−^ effector memory T cells in CD4^+^ T cells, but did not affect in CD8^+^ T cells. This suggests that L.E.M.-stimulated BMDCs enhance T-cell IFN-γ production and transform CD4^+^ T cells and CD8^+^ T cells into effector memory T cells.

### L.E.M. extract activates BMDMs and induces M1-like macrophages

**Fig. 3:**
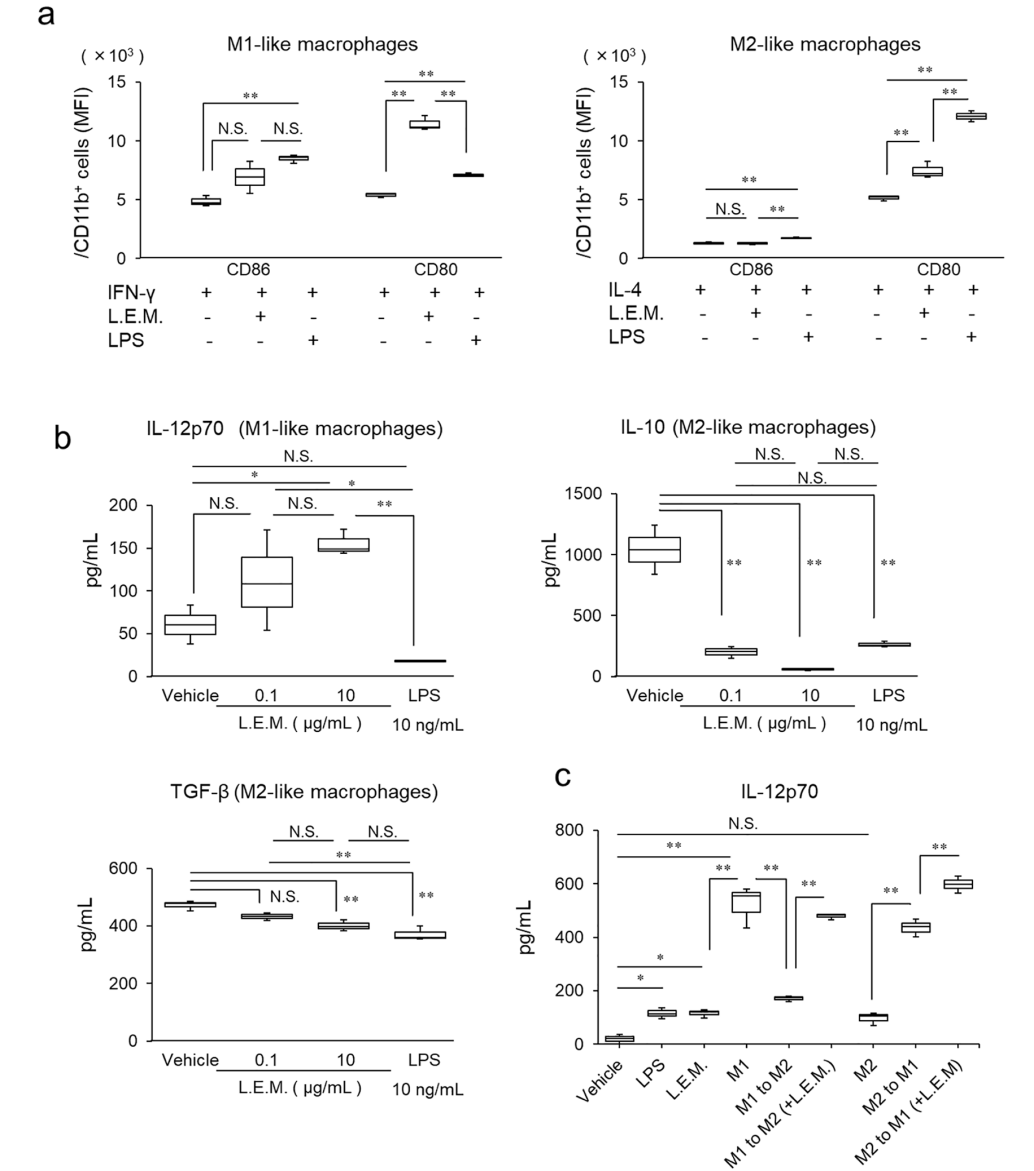
L.E.M. extract activates BMDMs and induces M1-like macrophages. L.E.M. extract activates BMDMs and induces M1-like macrophages. (**a**) IFN-γ (20 ng/mL) or IL-4 (20 ng/mL) were added to BMDMs to induce M1-like macrophages or M2-like macrophages, respectively, then stimulated with 10 µg/mL L.E.M. extract or 10 ng/mL LPS (a positive control). After culturing for three days, the expression levels of CD86 and CD80 were analyzed by flow cytometry (N = 3 per group). (**b**) Similarly, M1-like macrophages and M2-like macrophages were induced and stimulated with 0.1 or 10 µg/mL L.E.M. extract. After culturing for three days, the macrophages were stimulated with 10 ng/mL LPS for 6 h, and the level of IL-12p70 production in the M1-like macrophage culture supernatant and level of IL-10 and TGF-β production in the M2-like macrophage culture supernatant were measured by ELISA assay (N = 3 per group). (**c**) Similarly, M1-like macrophages and M2-like macrophages were induced, IL-4 (20 ng/mL) was added to M1-like macrophages to induce M2-like macrophages, and the cells were cultured for three days after stimulating with 10 µg/mL L.E.M. extract (M1 to M2). IFN-γ (20 ng/mL) was added to M2-like macrophages to induce M1-like macrophages, whereas 10 µg/mL L.E.M. extract was added to stimulate the cells, which were then cultured for three days (M2 to M1). After culturing, each set of cells were stimulated for 6 h with 10 ng/mL LPS, and the level of IL-12p70 production in the culture supernatant was measured by ELISA analysis (N = 3 per group). The median value is shown in the figure. The data are representative of two independent experiments and were statistically analyzed by one-way ANOVA (*, p < 0.05; **, p < 0.01; N.S., not significant)

Macrophages function as APCs but do not exhibit a single property. Rather, macrophages can be broadly categorized into two subsets: M-1like macrophages that release cytokines such as IL-12 and are responsible for T-cell activation and tumor elimination; and M2-like macrophages that release immunosuppressive cytokines such as IL-10 and TGF-β, and are involved in tissue repair and tumor growth [26, 27]. Therefore, to investigate the effect of L.E.M. extract on each type of macrophage, BMDMs were induced, then L.E.M. extract or LPS was added when inducing M1-like macrophages by IFN-γ and M2-like macrophages by IL-4. According to flow cytometry, the expression level of CD86 in M1-like macrophages and M2-like macrophages was not significantly affected by the addition of L.E.M. extract, whereas the expression level of CD80 was significantly increased (Fig. 3a). Conversely, when LPS was added, the expression levels of CD86 and CD80 were significantly increased in each type of macrophages. ELISA measurement in the supernatant of L.E.M.-stimulated macrophages showed that L.E.M. extract increased the production of IL-12 in a dose-dependent manner in M1-like macrophages, but suppressed the production of the IL-10 and TGF-β in M2-like macrophages (Fig. 3b). Conversely, LPS-added macrophages did not produce a significant difference in IL-12 production compared to the vehicle, but significantly suppressed IL-10 and TGF-β production.

M1 and M2 macrophages are reversible depending on the surrounding environment [28]. Therefore, we examined the effect of L.E.M. extract on the plasticity of macrophages under the condition that M1-like macrophages change to M2-like macrophages or M2-like macrophages change to M1-like macrophages. When M2-like macrophages were induced by adding IL-4 after inducing M1-like macrophages, IL-12p70 production decreased compared to that in M1-like macrophages. Conversely, when L.E.M. extract was added, IL-12p70 production did not decrease but remained at approximately the same level as that in the original M1-like macrophages (Fig. 3c). Furthermore, when M1-like macrophages were induced by adding IFN-γ after inducing M2-like macrophages, IL-12p70 production was significantly higher than that in M2-like macrophages, whereas when L.E.M. extract was added, IL-12p70 production increased further (Fig. 3c). This suggests that L.E.M. extract directly activates macrophages, promotes the generation of M1-like macrophages, and preserves M1-like functions in an environment that induces M2-like macrophages.

### L.E.M. extract suppresses the reduction in the number, proportion, and activity of DCs in the spleen of cancer-bearing mice

**Fig. 4:**
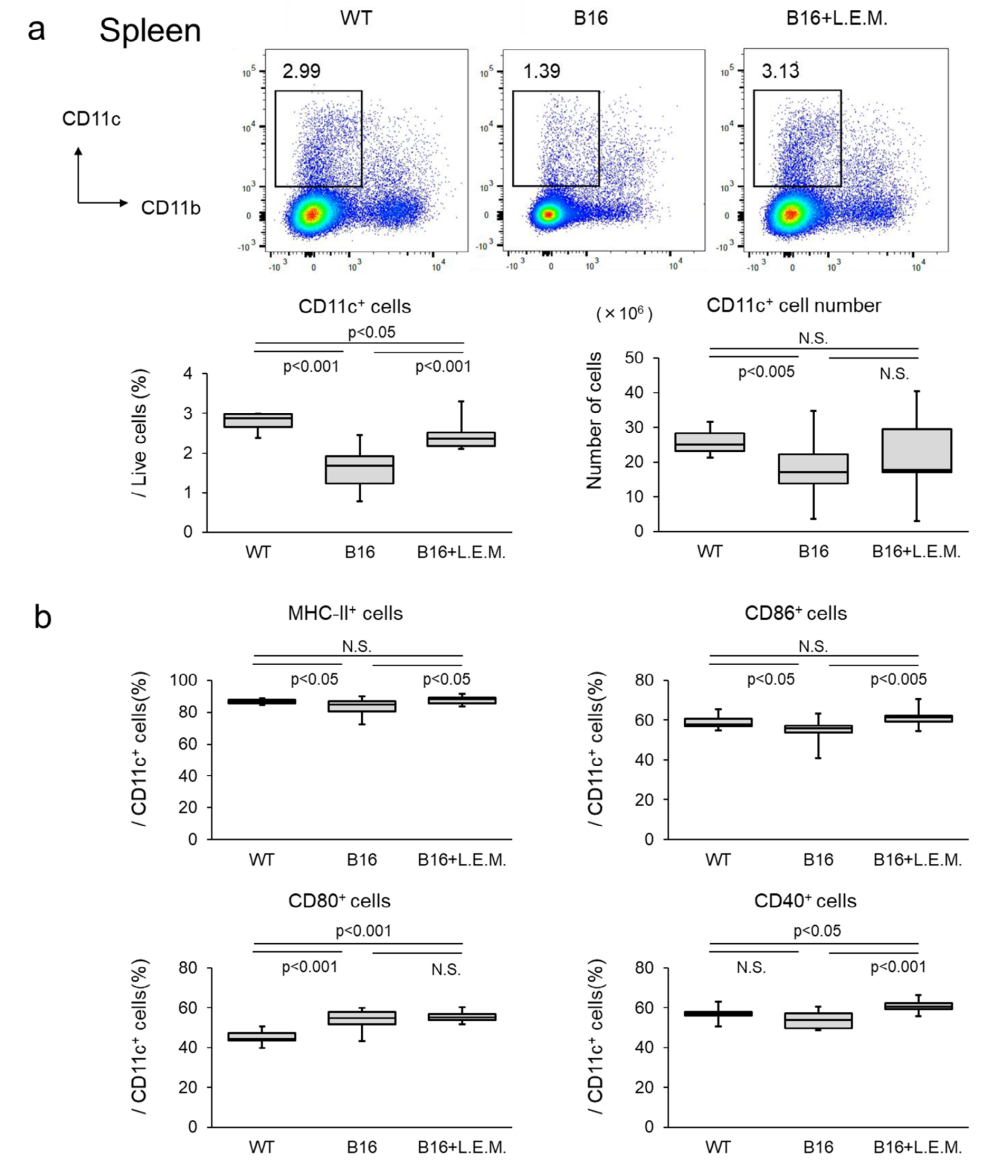
L.E.M. extract suppresses the attenuation of DCs activity in cancer-bearing mice L.E.M. extract suppresses the attenuation of dendritic cell activity in cancer-bearing mice. B16F10 cells (7.5 × 10^5^ cells) were injected subcutaneously into the left sole of the mice (WT, N = 8; B16 group, N = 15; B16-L.E.M. group, N = 15). From Day 7 after administering the tumor cells, the mice were orally administered 200 µL of PBS or L.E.M. extract daily at a dose of 2 g/kg. Mice that showed tumor metastasis from the left sole to the thigh after tumor cell administration were euthanized (B16 group, N = 2; B16-L.E.M. group, N = 2). On Day 28 after administration, the mice were euthanized, the tumor weight was measured, and the spleen was recovered. (**a**) Proportion of DCs in the spleen analyzed as CD11c^+^CD11b^−^ cells by flow cytometry. The figures show the percentage of CD11c^+^CD11b^−^ cells, as well as typical flow cytometry results. The median value is shown in the figure. Data were analyzed by Student’s t-test or Welch’s t-test (N.S., not significant). (**b**) Proportion of MHC-II^+^, CD80^+^, CD86^+^, and CD40^+^ cells in CD11c^+^ cells analyzed by flow cytometry. The median value is shown in the figure. The data are representative of two independent experiments and were statistically analyzed by Student’s t-test or Welch’s t-test (N.S., not significant)

Next, we studied the effect of L.E.M. extract on DCs and macrophages in vivo using cancer-bearing mice. First, B16F10 melanoma cells were administered into the footpad of mice, and L.E.M. extract was orally administered daily at a dose of 1 g/kg and 2 g/kg from Day 7 following the date of tumor cell administration. As previously reported, tumor growth was inhibited in response to the dose of L.E.M. extract intake [14] and in mice inoculated with GEM as a positive control for tumor regression (Supplementary Fig. 3). Therefore, we performed subsequent experiments at a dose of 2 g/kg, which is highly effective in suppressing tumors with L.E.M. extract. When DCs in the spleen were first analyzed by flow cytometry, the number and proportion of DCs in cancer-bearing mice were significantly lower than those in non-cancer-bearing mice (Fig. 4a). In the L.E.M. group, the proportion of DCs were significantly higher and the number tended to be higher than in the tumor-bearing model; therefore, we subsequently analyzed the activation of DCs. We observed that the proportion of MHC-II^+^ cells and CD86^+^ cells in DCs was significantly reduced in cancer-bearing mice compared to non-cancer-bearing mice. These decrease were significantly suppressed in the L.E.M. group (Fig. 4b). Furthermore, although no significant difference was observed in the proportion of CD40^+^ cells in the cancer-bearing mice compared to no-cancer-bearing mice, the L.E.M. group showed a significant increase compared to the tumor-bearing model. The proportion of CD80^+^ cells increased in cancer-bearing mice; however, L.E.M. extract intake had no effect. Therefore, we suggest that the number and proportion of DCs are reduced in the cancer-bearing state and that the activity level of DCs also decreases, resulting in a dysfunctional state. Furthermore, we suggest that L.E.M. extract suppresses or improves this dysfunctional state of DCs.

### L.E.M. extract suppresses the reduction in the proportion and activity of macrophages in the spleen of cancer-bearing mice

**Fig. 5:**
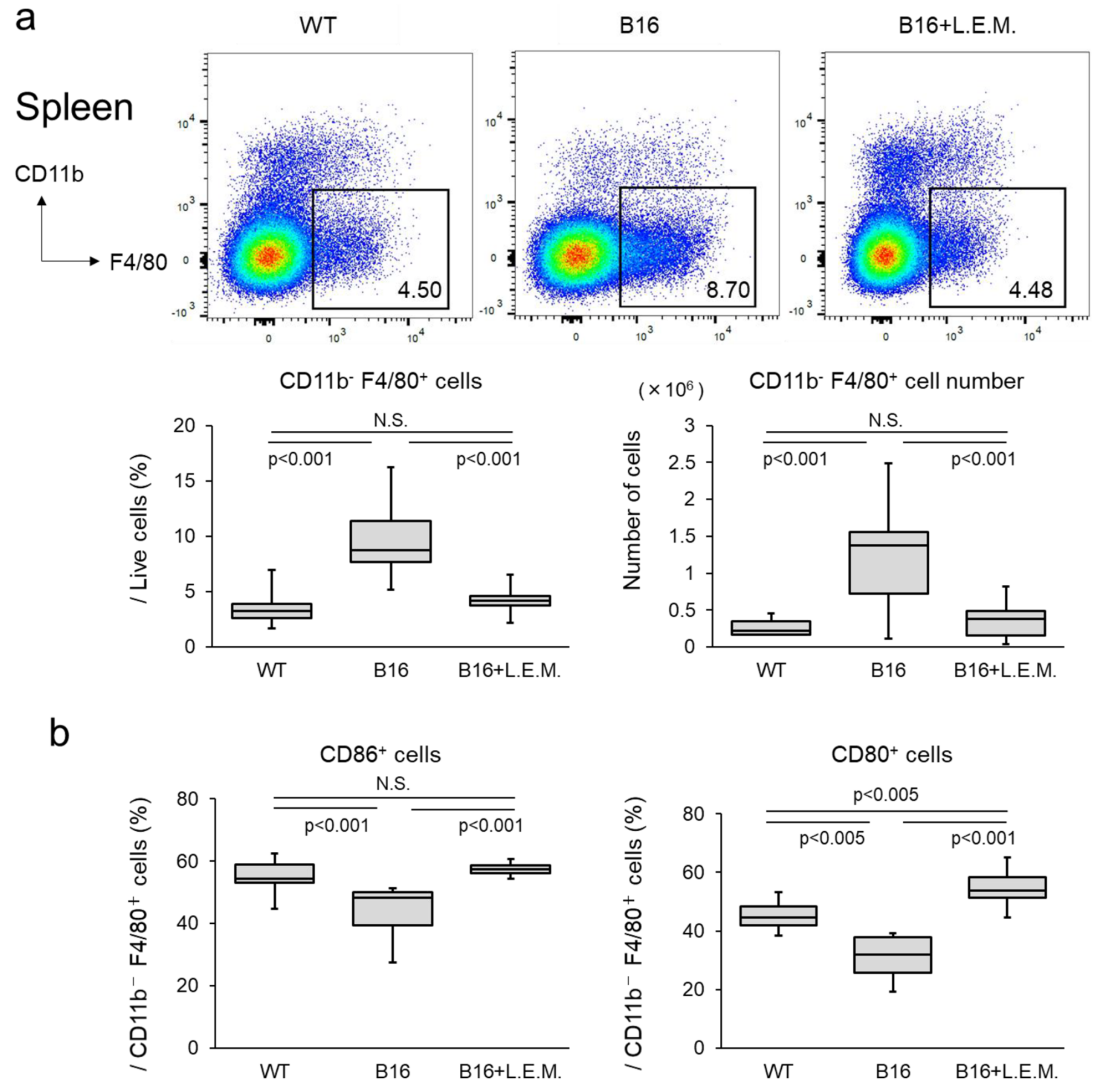
L.E.M. extract suppresses macrophage changes in cancer-bearing mice L.E.M. extract suppresses macrophage changes in cancer-bearing mice. B16F10 cells (7.5 × 10^5^ cells) were injected subcutaneously into the left sole of the mice (WT group, N = 8; B16 group, N = 15; B16-L.E.M. group, N = 15). From Day 7 after administering the tumor cells, the mice were orally administered 200 µL of PBS or L.E.M. extract daily at a dose of 2 g/kg. Mice that showed tumor metastasis from the left sole to the thigh after tumor cell administration were euthanized (B16 group, N = 2; B16-L.E.M. group, N = 2). On Day 28 after administration, the mice were euthanized, the tumor weight was measured, and the spleen was recovered (WT group, n = 5; B16 group, n = 6; B16-L.E.M. group, n = 4). (**a**) Percentage of macrophages in the spleen measured by CD11b and F4/80 and analyzed by flow cytometry. Figures show the percentage of CD11b^−^F4/80^+^ cells, as well as the typical flow cytometry results. The data are representative of two independent experiments and were statistically analyzed by Student’s t-test or Welch’s t-test (N.S., not significant). (**b**) Proportion of CD86^+^ cells and CD80^+^ cells in CD11b^−^F4/80^+^ cells analyzed by flow cytometry. The median value is shown in the figure. The data are representative of two independent experiments and were statistically analyzed by Student’s t-test or Welch’s t-test (N.S., not significant)

Next, we analyzed macrophages. The proportion and number of CD11b^−^F4/80^+^ macrophages was significantly higher in the cancer-bearing state than in the non-cancer-bearing state, but was then significantly decreased in the L.E.M. group (Fig. 5a). Therefore, we then focused on the activation of CD11b^−^F4/80^+^ cells. The proportion of CD80^+^ cells and CD86^+^ cells in CD11b^−^F4/80^+^ cells were significantly lower in cancer-bearing mice than in non-cancer-bearing mice (Fig. 5b). This decrease was significantly suppressed in the L.E.M. group. These results suggest that macrophages with poor activation are increased in the cancer-bearing state, and that L.E.M. extract administration then suppresses the increase in these macrophages.

## Discussion

To efficiently eliminate cancer, it is important to understand not only immune activation aspects such as recognition and elimination of cancer cells by the immune system, but also the immunosuppressive mechanism by which cancer escapes attack from immune cells. Specifically, DCs and macrophages are important APCs for complex immune regulation, which includes the activation of tumor antigen-specific T cells and induction of immunosuppression [29, 30], and are considered key factors contributing to the antitumor effect of L.E.M. extract. Here, we identified the role of DCs and macrophages in the L.E.M. extract mechanism, and confirmed that L.E.M. extract enhances the antigen-presenting capacity of cells by acting on these APCs.

In this study, we present a unique function of L.E.M. extract that involves direct activation of BMDCs, enhanced IL-12 production, induction of effector memory T cells by activated DCs, and enhanced IFN-γ production by CD8^+^ T cells. Studies using LPS have shown that IL-12 production from BMDCs induces a Th1 response and is strongly involved in the induction of antigen-specific CTLs [31]. Moreover, IL-12 induces IFN-γ production by CD8^+^ T cells [32], and enhances T-cell proliferation by directly acting on CD44^low^CD8^+^ T cells during antigen stimulation to increase CD44 expression [33]. Furthermore, IL-12 is involved in long-term immunity by regulating the expression of the Bcl-3- and Bcl-2-related genes responsible for anti-apoptotic action through the phosphorylation of STAT4 [34]. In this study, L.E.M. extract had a stronger ability to induce IL-12 production than LPS, which suggests that L.E.M. extract is useful for the efficient induction of CTL by regulating the function of CD8^+^ T cells.

However, from the perspective of immunosuppression, immunosuppressive factors such as IL-6, TGF-β, and IL-10 produced from tumor cells attenuate the activation level of DCs [35–37]. Even in terms of T-cell activation, because IL-6 KO mice exhibit greater dendritic cell-mediated T-cell activation than wild-type mice [38], the differentiation and maturation of DCs are suppressed by cytokines such as TGF-β, IL-6, and IL-10 produced by tumor cells; thus, the induction of tumor antigen-specific CTLs and antitumor immune response are likely suppressed. We found that L.E.M. extract intake managed the attenuated levels of dendritic cell activation in the cancer-bearing state. Consistent with this finding, it has been reported that L.E.M. extract intake suppresses TGF-β and IL-6 in the blood, which increases in the cancer-bearing state [15]. Additionally, it has also been reported that syringic acid and vanillic acid in L.E.M. extract suppress immune-mediated hepatitis via modulation of IL-6 in a mouse model of concanavalin A-induced liver injury [24], and that vanillic acid inhibits IL-6 secretion in LPS-stimulated mouse peritoneal macrophages [23]. Therefore, L.E.M. extract not only directly activates DCs but also contributes to the induction of anti-tumor responses through a complex mechanism of maintaining dendritic cell function by suppressing the production of immunosuppressive cytokines such as TGF-β and IL-6.

The results of this study suggest that L.E.M. extract acts on macrophages as well as DCs. In macrophages, circulating monocytes are mobilized into tissues then differentiated into macrophages, where they express polarized functional characteristics through the surrounding environment such as the influence of microorganisms and cytokines [28]. L.E.M. extract directly activated BMDMs and induced M1-like macrophages that produced the inflammatory cytokine IL-12. Furthermore, we confirmed that L.E.M. extract preserves M1-like macrophages under the condition of converting M1-like macrophages into M2-like macrophages that produce the immunosuppressive cytokines TGF-β and IL-10. This suggests that L.E.M. extract may be able to induce M1-like macrophages even in an immunosuppressive environment in which M2-like macrophages are induced, such as cancer-bearing mice. In fact, the culture supernatant of B16F10 cells (melanoma conditioned medium) transforms macrophages into M2-like macrophages [39]; however, when culturing BMDMs with melanoma conditioned medium derived from B16F10 cells, the addition of L.E.M. extract induced M1-like macrophages (Supplementary Fig. 4).

In vivo experiments suggested that the number and proportion of CD11b^−^F4/80^+^ macrophages in the spleen were significantly higher in cancer-bearing mice than in wild-type mice, and these changes were suppressed in the L.E.M. group. CD11b^−^F4/80^+^ cells are tissue macrophages present in the splenic red pulp (termed red pulp macrophages), and are involved in the removal of aged red blood cells and the metabolism of iron [40]. It is possible that red pulp macrophages are positive for CD163 and CD206 expressed on M2-like macrophages and tumor-associated macrophages [41]. Although little is known about the role of red pulp macrophages in cancer, in breast cancer-derived 4T1 cell-administered mice, the depletion of spleen macrophages reduces the expression of bone morphogenic protein 4 and inhibits red blood cell formation in the splenic red pulp, thereby suppressing tumor growth [42]. Furthermore, macrophage progenitor cells accumulated in the splenic red pulp of a cancer-bearing mouse model with genetically developed lung adenocarcinoma, and macrophages were mobilized in the tumor by tumor-derived factors CCL2 and CSF-1 [43]. Furthermore, the administration of anti-F4/80 antibodies to deplete macrophages delays tumor growth in AE17 cancer-bearing mice derived from splenomegaly [43]. Interestingly, in our study, the proportion of CD11b^−^F4/80^+^ cells in the spleen showed a positive correlation with tumor weight, whereas the proportion of CD86^+^ cells and CD80^+^ cells in CD11b^−^F4/80^+^ cells showed a negative correlation with tumor weight (Supplementary Fig. 5). This suggests that poorly activated CD11b^−^F4/80^+^ cells accumulate in the spleen in cancer-bearing mice, and may be mobilized locally in the tumor to attenuate the antitumor immune response. Furthermore, as L.E.M. extract intake suppresses these changes in CD11b^−^F4/80^+^ cells in the spleen, we suggest that L.E.M. extract may preserve the antitumor response by improving the cancer-induced dysfunctional state of macrophages in the spleen.

APCs are activated by the binding of various extrinsic factors such as fungi and molds to receptors such as toll-like receptors (TLRs) and dectins. For example, TLR2 binds to peptidoglycan and zymosan, and TLR4 activates APCs by binding to LPS [44]. Furthermore, Dectin-1 activates APCs via β (1 → 3)-glucan binding [45]. Lentinan, an antineoplastic preparation produced from β-glucan derived from shiitake mushrooms, has been shown to activate DCs via the stimulation of TLR2 and TLR4; in a malaria infection model, lentinan enhanced IL-12 and IFN-γ production in the spleen as well as the Th1 immune response [46]. Since *lentinula edodes* has been reported to contains β-glucan [47], L.E.M. extract may activate APCs via TLR2, TLR4, and Dectin-1. However, as L.E.M. extract is a preparation containing various components, it is possible that multiple components stimulate various receptors and work in a coordinated manner to activate APCs. For example, it has been reported that vanillic acid stimulates the STING/TBK1/IRF3 pathway in macrophages and promotes polarization of macrophages to an M1-like phenotype, and that intraperitoneal administration of vanillic acid to a 4T1 breast cancer cell inoculated mouse model inhibited tumor growth and changed macrophages in the tumor to an M1-like phenotype [48]. Considering the concentration of vanillic acid in L.E.M. extracts, it is suggested that there are other components in L.E.M. extracts that polarize macrophages into an M1-like phenotype, but the effect of vanillic acid described above could explain part of the effect of L.E.M. on APCs revealed in this study. We anticipate that further research into L.E.M. extract components will elucidate their role in the activation of different APCs.

## Conclusions

In this study, we revealed that L.E.M. extract directly activates the APCs involved in activating immune function and induces T-cell activation. L.E.M. extract also suppresses the attenuation of APCs that occurs in a cancer-bearing state, and may ultimately suppress cancer progression. As the key function of L.E.M. extract is to regulate the function of APCs, L.E.M. extract maintains and regulates the body’s immune function through APCs; thus, this extract is expected to be a useful treatment not only for cancer but also for infectious diseases.

### Electronic supplementary material

Below is the link to the electronic supplementary material.

Supplementary Material 1: Supplementary Figures and Table.

## Data Availability

The datasets generated during and/or analysed during the current study are available from the corresponding author on reasonable request.

## Abbreviations

APCs: antigen-presenting cells
BM: bone marrow
BMDCs: bone marrow-derived DCs
BMDMs: bone marrow-derived macrophages
CTL: cytotoxic T lymphocyte
DCs: dendritic cells
L.E.M.: *Lentinula Edodes Mycelia*
LPS: lipopolysaccharide
MACS: magnetic separation system
MHC: major histocompatibility complex
HPLC: high performance liquid chromatography
PBS: phosphate buffered saline
GEM: gemcitabine
TLRs: toll-like receptors
