# Supplementary material for: Lentinula Edodes Mycelia extract regulates the function of antigen-presenting cells to activate immune cells and prevent tumor-induced deterioration of immune function
Source: BMC Complement Med Ther. 2023 Aug 8;23:281. doi: 10.1186/s12906-023-04106-5 (PMC10408224; doi:10.1186/s12906-023-04106-5)
Supplement: Supplementary file 1 — Supplementary Material 1: Supplementary Figures and Table. [file 12906_2023_4106_MOESM1_ESM.docx]

**Supplemental Material**

**
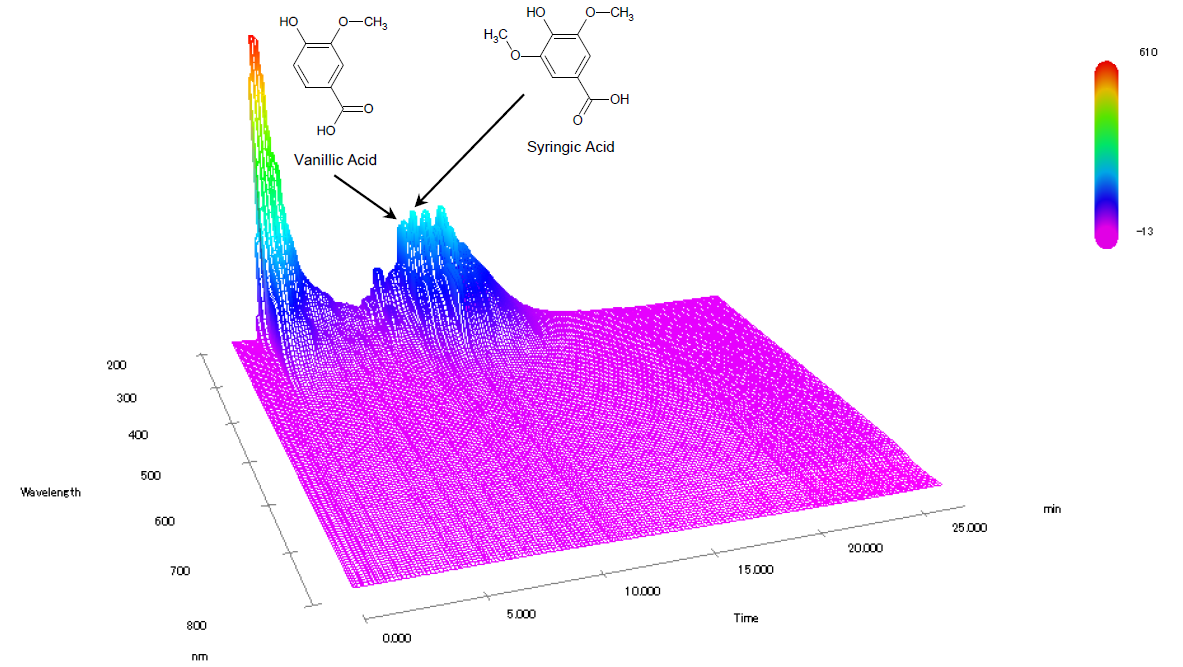
**

**Supplementary Figure 1 Chemical profile of L.E.M. extract obtained by High performance liquid chromatography (HPLC)**

Chemical profile obtained by high performance liquid chromatography (HPLC) of L.E.M. extract. HPLC was performed using a HPLC prominence (Shimazu, Kyoto, Japan). The conditions for HPLC analysis are as follows: columns; Atlantis T3 column (3 μm, 150 mm × 3 mm) (Waters, Milford, MA, USA), column temperature; 40°C, flow rate; 0.5 mL/min, injection volume; 1.0 μL, wavelength; 190-800 nm (photo diode array) ; mobile phase; a mixture of water (A) and acetonitrile (B). The eluent gradients were set as A-B (vol/vol) from 90:10 to 5:95. Syringric acid was detected at 274 nm (retention time; 9.786 min) and vanillic acid at 291 nm (retention time; 9.350 min).

**
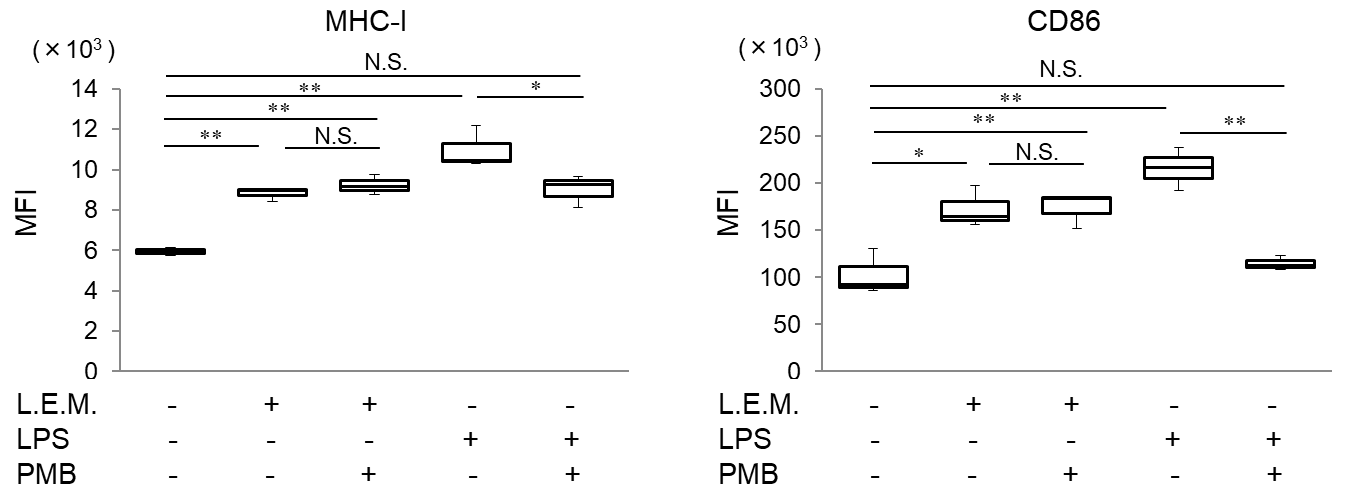
**

**Supplementary Figure 2 Investigation of the effects of endotoxin contamination of L.E.M. extracts**

Polymyxin B sulfate (PMB) was added to BMDCs to assess the potential for endotoxin contamination of L.E.M. extract.

When BMDCs were stimulated with either 10 μg/mL L.E.M. extract or 10 ng/mL LPS (positive control), 5 μg/mL PMB was added and incubated for 12 hours. Expression levels of MHC-I and CD86 were analyzed by flow cytometry (N = 3 per group). Data were obtained from one experiment and statistically analyzed by one-way ANOVA (*, p < 0.05; **, p < 0.01; N.S. not significant).

**
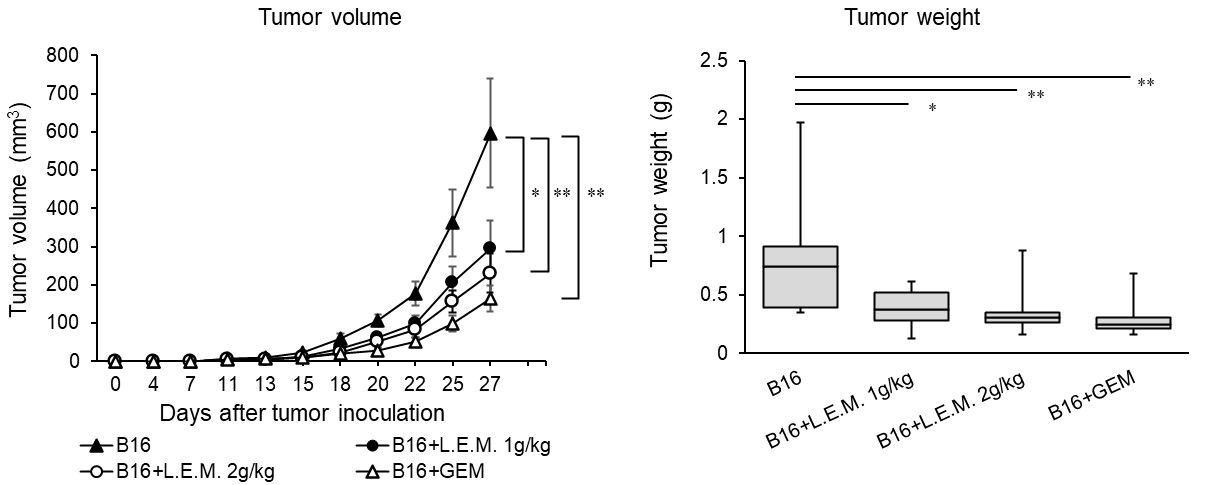
**

**Supplementary Figure 3** **Tumor volume and weight changes after L.E.M. extract intake**

Tumor volume and weight changes caused by L.E.M. extract intake. B16F10 cells (7.5×10^5^ cells) were injected subcutaneously into the left sole of the mice, and from Day 7 after administering the tumor cells, the mice were orally administered 200 μL of PBS or L.E.M. extract daily at a dose of 1 g/kg or 2 g/kg. To evaluate the degree of tumor regression in this study, 120 mg/kg gemcitabine was administered intraperitoneally twice on days 10 and 17 as a positive control. Starting from the date of intake, the tumor volume was measured twice per week. On Day 28 after administration, the mice were euthanized and the tumor weight was measured. These figures show the changes in tumor volume and the tumor weight on Day 28 (N= 10-14). Tumor volume data are shown as mean±SEM. Data are representative of two independent experiments, and tumor volume data on the date of final measurement were analyzed by one-way ANOVA and Tukey-Kramers post-hoc tests (*, p < 0.05; **, p < 0.01; N.S., not significant).. The median values are shown for the median tumor weight data, and the data were analyzed by one-way ANOVA and Tukey-Kramers post-hoc tests (*, p < 0.05; **, p < 0.01).


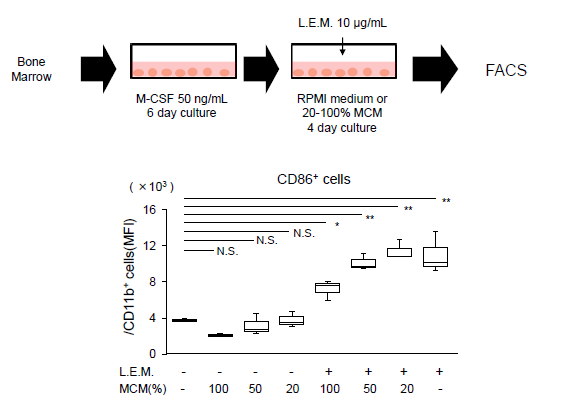


**Supplementary Figure 4** **L.E.M. extract maintains macrophage activity under culture in tumor culture supernatant**

L.E.M. extract preserves macrophage activity under culture in tumor culture supernatant. B16F10 cells were cultured in RPMI medium for four days and the culture supernatant was collected (100% MCM: melanoma conditioned medium). This was diluted with fresh RPMI medium to produce 20% and 50% MCM. BMDMs were cultured in RPMI medium or the prepared MCM for four days. L.E.M. extract was added at 10 μg/mL and the expression of CD86^+^ cells in CD11b^+^ cells was evaluated by mean fluorescence intensity via flow cytometry (N = 3 per group). The median value is shown in the figure. The data are representative of two independent experiments and were statistically analyzed by one-way ANOVA (*, p < 0.05, compared to control; **, p < 0.01, compared to control; N.S., not significant, compared to control).


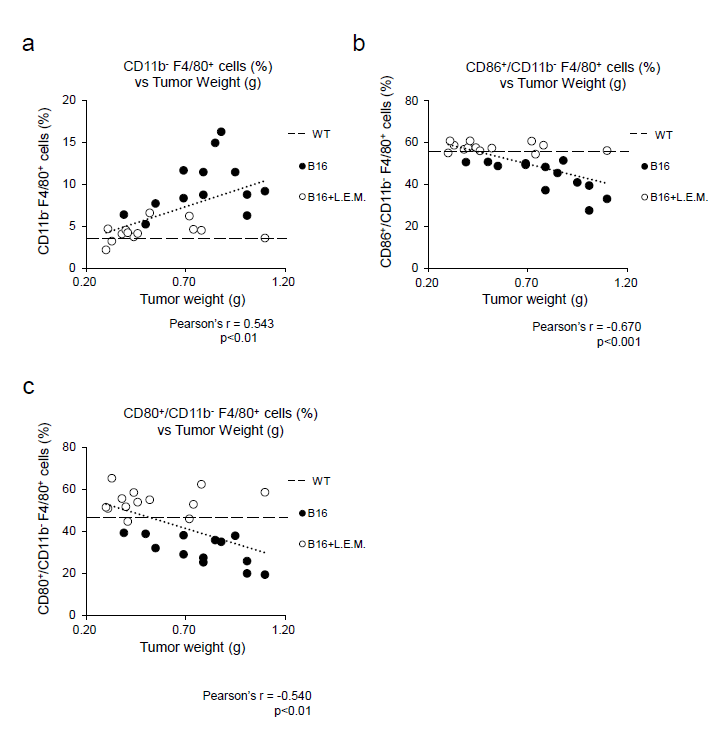


**Supplementary Figure 5 Correlation between macrophages and tumor weight**

Correlation between macrophages and tumor weight. (a) Correlation between the proportion of CD11b^-^F4/80^+^ cells and tumor weight in the spleen of cancer-bearing mice administered with B16F10 cells (N=13). Dotted line shows the mean value of the proportion of CD11b^-^F4/80^+^ cells in the wild-type group. (b) Correlation between the proportion of CD86^+^ cells in CD11b^-^F4/80^+^ cells and tumor weight (N = 13). Dotted line shows the mean value of the proportion of CD11b^-^F4/80^+^ cells in the wild-type group. (c) Correlation between the proportion of CD80^+^ cells in CD11b^-^F4/80^+^ cells and the tumor weight (N = 13). Dotted line shows the mean value of the proportion of CD11b^-^F4/80^+^ cells in the wild-type group. We also calculated the Pearson correlation coefficient (r) and the probability (p value) of obtaining the test statistic (t value) according to the t distribution.

**Supplementary Table 1** **Monoclonal antibodies used for flow cytometry analysis**

| Antibody | Fluorochrome | Clone | Manufacture |
| --- | --- | --- | --- |
| CD4 | PerCP | RM4-5 | BioLegend |
| CD8α | PE-Cy7 | 53-6.7 | BioLegend |
| CD11b | FITC | M1/70 | Thermo Fisher Scientific |
| CD11b | APC-Cy7 | M1/70 | BioLegend |
| CD11c | FITC | N418 | Miltenyi Biotec |
| CD11c | BV421 | N418 | BioLegend |
| CD40 | APC | 3/23 | BioLegend |
| CD44 | APC | IM7 | BioLegend |
| CD62L | PE | MEL-14 | BD Biosciences |
| CD80 | FITC | 16-10A1 | BioLegend |
| CD80 | PE | 16-10A1 | BioLegend |
| CD86 | APC | PO3 | Miltenyi Biotec |
| CD86 | PE-Cy7 | GL-1 | BioLegend |
| F4/80 | BV421 | BM8 | BioLegend |
| H-2D^b^ | PE | 28-14-8 | Thermo Fisher Scientific |
| I-A^b^ | FITC | AF6-120.1 | BioLegend |
| I-A^b^ | PE | AF6-120.1 | Thermo Fisher Scientific |

**Supplementary Figure 6 For editor and reviewers only**


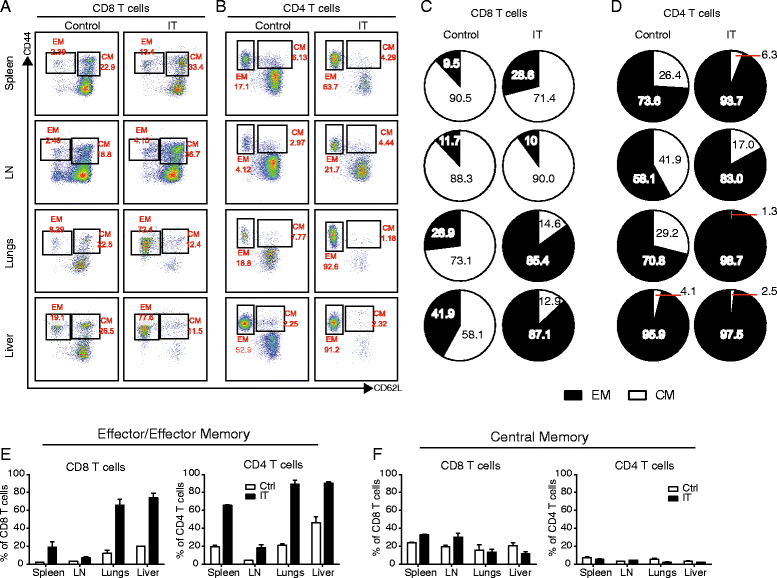


This figure is taken from the following document as a response to the editor's and reviewer's points.

Citation from:

Sckisel GD, Mirsoian A, Minnar CM, Crittenden M, Curti B, Chen JQ, Blazar BR, Borowsky AD, Monjazeb AM, Murphy WJ (2017) Differential phenotypes of memory CD4 and CD8 T cells in the spleen and peripheral tissues following immunostimulatory therapy, J Immunother Cancer 5:33. https://doi.org/10.1186/s40425-017-0235-4

　T cell memory phenotype differs in lymphoid and peripheral organs following immunotherapy. Mice were treated with anti-CD40/IL-2 immunotherapy and assessed for various immune parameters on day 12 of treatment in lymphoid (spleen or LN) or peripheral (lungs or liver) organs. a-b Representative dot plots of CD44 vs CD62L expression in CD8 (a) and CD4 (Foxp3-ve) (b) T cells in control and IT-treated mice. c-d Pie charts depicting central memory (white) vs effector/effector memory (black) frequency in the CD44high sub-population in CD8 (c) T cells and CD4 (d) T cells; frequencies of CD44high depicted within pie slices for given population. (e-f) Frequency of effector/effector memory (e) and central memory (f) CD8 (left panels) and CD4 (Foxp3-ve) (right panels) T cells in various organs from control or anti-CD40/IL2-treated mice. These data are representative of 4-5 independent experiments with 3 mice per group. Data are presented as mean ± SEM

**Supplementary Figure 7 For editor and reviewers only**


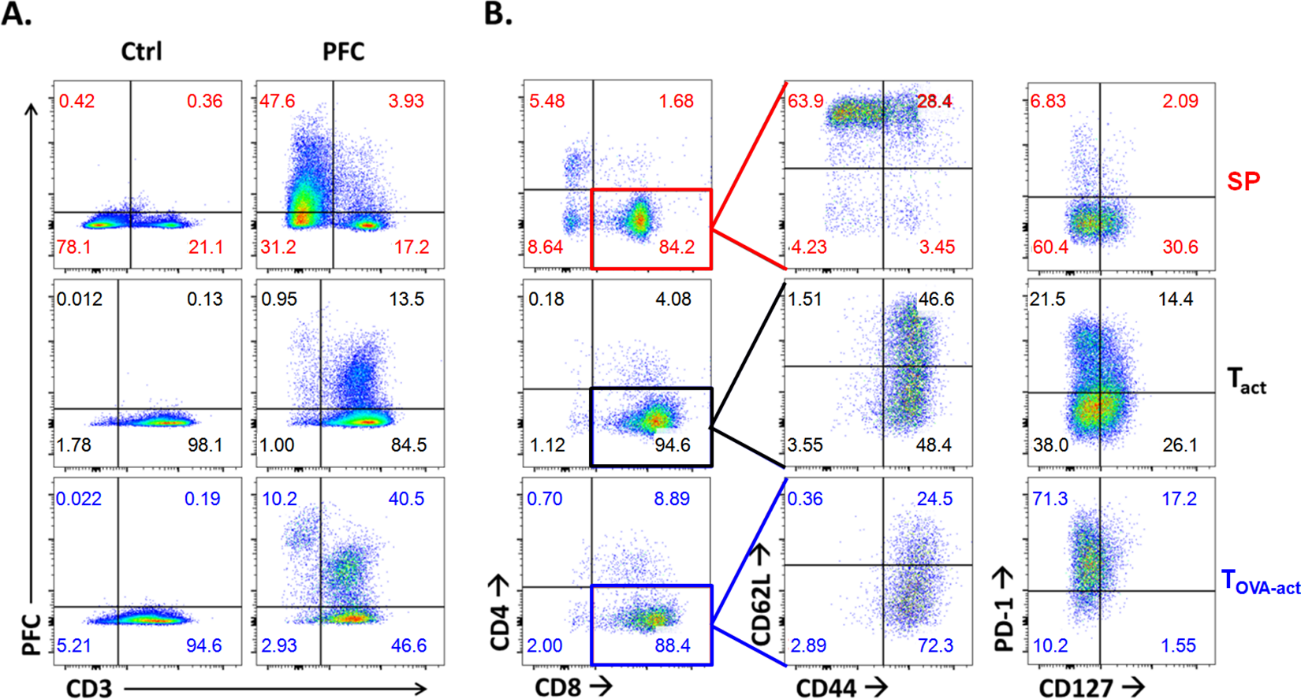


This figure is taken from the following document in response to the editor's and reviewer's points

Citation from:

Gonzales C, et al, In-Vivo Detection and Tracking of T Cells in Various Organs in a Melanoma Tumor Model by 19F-Fluorine MRS/MRI, PLoS One 11(10):e0164557, 2016; https://doi.org/10.1371/journal.pone.0164557

(A) PCF labelling of total splenocytes (SP), anti-CD3/anti-CD28 activated (Tact) and Ova-peptide activated (TOVA-act) splenocytes from OT-1 cells. (B) Phenotype of CD3+ T cells: CD3+CD8+ T cells were analyzed by flow cytometry for CD44, CD62L, CD127 and PD-1 expression. By this flow-cytometric analysis, the following phenotypes could be identified in the CD3+CD8+ T-cell population: CD62L+CD44- (naive T cells), CD62L+CD44+ (memory T cells) and CD62L-CD44+ (effector T cells). n = 3 for each group of stimulation. Data are representative of 3 independent experiments.
